# Correction: Clinical outcomes of breakthrough COVID-19 after booster vaccination in patients with systemic rheumatic diseases

**DOI:** 10.1136/rmdopen-2022-002279corr1

**Published:** 2022-03-30

Fragoulis GE, Karamanakos A, Arida A, *et al*. Clinical outcomes of breakthrough COVID-19 after booster vaccination in patients with systemic rheumatic diseases. *RMD Open* 2022;8:e002279. doi:10.1136/rmdopen-2022-002279

This article has been corrected since it was first published online. Maria G Tektonidou was incorrectly listed as Maria GG Tektonidou.

